# Faecal Occult Blood Point-of-Care Tests

**DOI:** 10.1007/s12029-018-0169-1

**Published:** 2018-09-20

**Authors:** Barbara Kościelniak-Merak, Branimir Radosavljević, Andrzej Zając, Przemysław J. Tomasik

**Affiliations:** 1 Department of Clinical Biochemistry, Institute of Pediatrics, Jagiellonian University Medical College, Wielicka St 265, 30-663 Cracow, Poland; 2 Institute of Chemistry in Medicine “Prof. dr Petar Matavulj”, Faculty of Medicine, University of Belgrade, Belgrade, Serbia; 3 Department of Pediatric Surgery, Jagiellonian University Medical College, Cracow, Poland

**Keywords:** Point-of-care, Immunochemistry, Occult blood, Stool sampling, Colorectal cancer, Screening

## Abstract

**Background:**

Early detection of colorectal cancer decreases the risk of mortality. Faecal occult blood tests (FOBT) are recognised as a useful tool for colorectal cancer screening. These non-invasive, rapid, and easy-to-carry assays are very often used as a point-of-care test and for self-testing. On the market, there are various types of FOB tests available, including chemical and immunochromatographic tests, which are based on different detection methods and differ in their sensitivity and specificity.

**Conclusions:**

Clinicians should be aware of the causes of false-negative and false-positive test results, which can vary depending on the test. Additionally, stool sampling bias may be a source of error and must be considered by the clinician. The current FOBT methods are subject to various interfering factors; items such as proper preparation of the patient prior to testing or the clinician’s knowledge of testing limitations are key in correct interpreting results. Novel technologies such as FOBT DNA tests, micro RNA tests, and biochips equipped with bacteria can indicate bleeding from the gastrointestinal tract and improve diagnostics process.

## Introduction

Faecal occult blood tests (FOBT) are used to detect blood in a stool which is not macroscopically visible [1, 2]. These tests are primarily performed as a first-line screening test for colorectal cancer (CRC), which is the third most common cancer in the world behind lung and breast cancer [2, 3]. According to statistics from the American Cancer Society (ACS), an estimated 140,250 new cases and 50,630 deaths from CRC are expected to occur in 2018. The risk of developing colorectal cancer is estimated as 1 per 22 (4.49%) for men and 1 per 24 (4.15%) for women, with case prognosis being strongly dependent on early detection of disease [4]. Therefore, the ACS recommends annual stool-based screening for persons over the age of 45 regardless of gender who have an average risk of CRC. Average risk is defined by the following patient characteristics: having no personal history of CRC, certain types of polyps, inflammatory bowel disease, or receiving radiation treatment to the abdomen or pelvic area to treat a prior cancer; no family history of colorectal cancer; and no evidence of confirmed or suspected hereditary colorectal cancer syndrome [5]. FOBT is a non-invasive, painless, inexpensive method that can be performed by the patients themselves at home [6].

There are currently three types of FOBTs based on different measurement methods: chemical tests, immunochromatographic tests, and DNA tests, which are not commonly available. These tests differ not only in the detection method but also in their susceptibility to cross-reactions and interfering factors [1, 7, 8]. Regardless of the test in use, clinicians should inform patients about contraindications. FOBTs should not be performed on patients experiencing menstruation, bleeding from haemorrhoids, or bleeding from the urinary tract due to the risk of contamination of stool with blood from these areas. Failure to follow these guidelines may lead to false-positive results. It should be also noted that the result of FOBT cannot be the basis for making clinical decisions. Positive results should be followed by an endoscopic examination [9, 10].

## Comparison of FOBTs

There are two types of chemical FOBT. The first is guaiac FOB (gFOB) testing, which is a qualitative assessment of the pseudo-peroxidase activity of haem from haemoglobin molecules found in faecal blood. Detection of blood in the stool occurs when blood-derived haem mediates the oxidation of guaiac acid by hydrogen peroxide into guaiac blue [7, 11]. The gFOB test is limited by a high risk of false-positives results [12]. Before performing a gFOB test, the patients should be instructed on how to properly prepare themselves [12, 13]. It is important that the patient avoids consumption of red meat along with certain vegetables and medicines for a minimum 2 to 7 days prior to the test [12, 14]. False-positive results may be caused by the presence of animal-derived haem, consumption of large amounts of green vegetables which may have chlorophyll-mediated pseudo-peroxidase activity and other foods (due to the naturally high levels of peroxidase) (broccoli, cauliflower, cantaloupe, carrots, squashes, figs, horseradish, grapefruit, melons, tomatoes, pumpkins, courgettes, etc.), or medicines including NSAID, vitamin C, and iron [13–15]. These requirements can be difficult for the patient and therefore the diagnostic specificity of the guaiac test is low, reaching only 50% [13].

An alternate to gFOB testing is 3,3′,5,5′-tetramethylbenzidine (TMB) testing. The TMB test is a one-stage test usually used as a pads (test papers) [15]. Paper soaked with the TMB indicator should be placed in a toilet bowl just after defaecation [16]. In this test, haem derived from free haemoglobin, lysed erythrocytes, or myoglobin catalyses the oxidation reaction of the indicator in an aqueous environment and triggers the test paper to change colour after 2 min into a green or blue colour [16]. While the performance of this test is simple and convenient for patients, the test only detects the blood present in the external layer of the stool. Similar to the gFOB test, TMB FOBTs are also vulnerable to interfering factors and the patient should exclude red meat, medications, green vegetables, and citrus fruit from the diet at least 3 days prior to testing [15, 16]. Furthermore, reading the results of the test is not objective as the patient must decide whether the colour change is adequate. This could be confusing and may lead to an improper interpretation of the test results, especially if the patient reads the test result from a distance.

The other groups of FOBTs are immunochemical tests (iFOBT or FIT—faecal immunochemical test), which detect a human globin, a protein that is a component of haemoglobin using specific antibodies [14, 17]. There are two types of iFOBT: qualitative, point-of-care tests and quantitative, automated tests [18]. Quantitative iFOB tests measure concentration of haemoglobin in a stool sample on an automated analyser. Although they provide detailed results, they can only be performed in a medical laboratory [18]. Qualitative iFOB tests, which are used at point of care, are usually prefabricated cassettes where results are read by visual inspection. A coloured band appears in the test patch if the haemoglobin is present in the faeces. It is important to remember that tests supplied by different manufacturers have different sensitivities to haemoglobin concentration in the stool [19]. After defaecation, samples should be collected from several places of the stool including inner parts and mixed with buffer prior to application onto the testing area of the cassette [19]. The main advantage of the iFOB method is the lack of interference from animal haemoglobins or fruit and vegetable compounds, allowing the patients to maintain their normal diet. Also, patients do not need to discontinue drugs which commonly interfere with the gFOB method [14].

Nowadays, researchers are continuously working on the development of new technologies which will significantly improve the efficiency and accuracy of FOB detection. Recently, Mimee et al. have described an ingestible sensor equipped with genetically engineered bacteria which can indicate bleeding in the gastrointestinal tract. Authors reported that biomolecular monitoring of the gut might be precise and faster than other laboratory methods [20].

The knowledge about test limitation and possible interferences is a key point for proper screening [12]. Generally, the iFOB test is more specific than gFOB test and not subject to the interfering factors like gFOBT [14]. Studies which compared the specificity and sensitivity of the commercially available FOB tests have demonstrated that iFOB testing maintains an advantage over the available chemical tests in the ability to detect CRC [21]. A recent US study which involved 5841 patients has shown that iFOB tests were able to detect 81.8% of individuals with invasive CRC, while the gFOB test detected only 64.3% of CRC cases. Additionally, the specificity of these tests for CRC detection was 96.9% for iFOB testing and 90% for gFOB testing [22]. In Germany, six different commercially available iFOBTs were compared with the gFOBT. The researchers examined 1319 patients with risk for CRC, with the iFOBTs demonstrating 97% specificity and gFOBT 95%. The summary diagnostic sensitivity was 50% for the iFOBTs and 30% for the gFOB test [23]. A study performed by Cruz-Correa et al. compared gFOB and TMB testing and demonstrated that while the guaiac test is characterised by both higher specificity and sensitivity, patients preferred the test with TMB (tissue pad), considering it to be more comfortable and easier to perform [24]. Comparing iFOB testing, sampling requires fewer episodes than chemical testing, but due to the modern construction of the toilet bowls where the faeces drop directly into the drain, the correct sample collection is very difficult. The stool sample may be contaminated with residues of previous defaecations and urinations, chemical toilet cleaners, disinfectants, and fragrances [25]. The typical method of proper stool collection can be uncomfortable, as the excrements must be collected using a plastic container. Specially designed containers for stool collection that are more user-friendly are also available [24].

Doctors should also remember that chemical FOB tests could be positive in the cases of bleeding from all parts of GI tract, whereas the diagnostic value of iFOBT is limited to the detection of bleeding from the lower gastrointestinal tract since globin from the upper GI is readily degraded by digestive proteolytic enzymes [26]. This was confirmed by a study which demonstrated that up to 100 mL of ingested blood went undetected by some immunochemical methods but was detected by gFOBT [26–28]. There have been numerous cases of massive bleeding from the stomach and duodenum, with tarry stools, where the iFOBT gave negative results due to digestion of the globin during passage through the gastrointestinal tract. In the case of tarry stools (after excluding the therapy with coal, consumption of berries, swallowing blood from nosebleeds, etc.) and the negative iFOBT, the upper gastrointestinal bleeding should be suspected [16]. Moreover, iFOBTs are not clinically specific to CRC. Non-neoplastic and benign pathologies may also bleed and give positive test results [28, 29].

## Other Alternative and Non-invasive Tests for CRC Screening

Several promising new technologies for CRC detection are currently in development. Multitarget faecal DNA and RNA assays are novel but are currently limited by high costs [10, 30]. These tests are based on detection of key mutations occurring during early stages of colon carcinogenesis including K-Ras, adenoma polyposis coli (APC), and p53 as well as epigenetic changes like a microsatellite instability. Furthermore, the second generation of stool DNA tests include analysis of methylated genes for vimentin, secreted frizzled-related protein 2 (SFRP2), bone morphogenetic protein 3 (BMP3), N-Myc downstream-regulated gene 4 protein (NDRG4), and tissue factor pathway inhibitor 2 (TFPI2) [29–34]. Recently, studies have shown that the DNA marker panels have a diagnostic sensitivity for detection of CRC of 92.3% and for precancerous lesions of 42.4%. Diagnostic specificity for these panels ranges from 86.6 and 94.9% [35]. These findings demonstrate an advantage for this test over colonoscopy in detecting ascending colon lesions. The researchers who carry out these studies plan to reduce DNA test costs and implement them as point-of-care diagnostics as a lab-on-chip or microarrays. A novel test involving detection of faecal microRNAs, a group of 18–25 nucleotide non-coding RNA molecules which regulate gene expression, is also in development. According to authors, this assay is an effective screening method, which may detect the presence of adenomas, advanced adenomas, and CRC with 62%, 73%, and 78% sensitivities respectively [36].

## Summary

The currently recommended FOBT for CRC screening is the iFOB test, performed annually. This test is, however, a preliminary examination and the diagnosis of colon cancer requires additional tests such as colonoscopy or sigmoidoscopy with a collection of a biopsy section for further pathomorphological and genetic examinations. In the case of bleeding from the upper gastrointestinal tract, there is currently a lack of high-sensitivity assays, although such bleeding can be detected by guaiac or TMB tests. These tests are sensitive to numerous interfering substances, and if there is a suspicion of bleeding from the upper or lower gastrointestinal tract, the results should be confirmed by gastroscopy and/or colonoscopy. Due to the wide availability of various kinds of FOBTs, physicians should pay attention to which tests they recommend to patients and what method is used when patients report results of self-testing. In the case of gFOB and TMB FOB tests, the clinician should discuss diet with the patient because diet is the main source of false-positive results. Also, the sampling procedures using commercially available devices should be presented to the patient. Developing new screening tests for CRC may improve the efficiency of its detection and therefore significantly limit mortality. Non-invasive testing may be a first-line screening option for asymptomatic individuals at average risk for CRC.
